# Pulmonary blood volume estimation in mice by magnetic particle imaging and magnetic resonance imaging

**DOI:** 10.1038/s41598-021-84276-9

**Published:** 2021-03-01

**Authors:** Michael Gerhard Kaul, Tobias Mummert, Matthias Graeser, Johannes Salamon, Caroline Jung, Enver Tahir, Harald Ittrich, Gerhard Adam, Kersten Peldschus

**Affiliations:** 1 Department of Diagnostic and Interventional Radiology and Nuclear Medicine, University Medical Center Hamburg-Eppendorf, Martinistrasse 52, 20246 Hamburg, Germany; 2 Institute for Biomedical Imaging, Hamburg University of Technology, Schwarzenbergstraße 95C, 21073 Hamburg, Germany

**Keywords:** Cardiovascular biology, Blood flow, Diagnostic markers, Imaging techniques and agents

## Abstract

This methodical work describes the measurement and calculation of pulmonary blood volume in mice based on two imaging techniques namely by using magnetic particle imaging (MPI) and cardiac magnetic resonance imaging (MRI). Besides its feasibility aspects that may influence quantitative analysis are studied. Eight FVB mice underwent cardiac MRI to determine stroke volumes and anatomic MRI as morphological reference for functional MPI data. Arrival time analyses of boli of 1 µl of 1 M superparamagnetic tracer were performed by MPI. Pulmonary transit time of the bolus was determined by measurements in the right and left ventricles. Pulmonary blood volume was calculated out of stroke volume, pulmonary transit time and RR-interval length including a maximal error analysis. Cardiac stroke volume was 31.7 µl ± 2.3 µl with an ejection fraction of 71% ± 6%. A sharp contrast bolus profile was observed by MPI allowing subdividing the first pass into three distinct phases: tracer arrival in the right ventricle, pulmonary vasculature, and left ventricle. The bolus full width at half maximum was 578 ms ± 144 ms in the right ventricle and 1042 ms ± 150 ms in the left ventricle. Analysis of pulmonary transit time revealed 745 ms ± 81 ms. Mean RR-interval length was 133 ms ± 12 ms. Pulmonary blood volume resulted in 177 µl ± 27 µl with a mean maximal error limit of 27 µl. Non-invasive assessment of the pulmonary blood volume in mice was feasible. This technique can be of specific value for evaluation of pulmonary hemodynamics in mouse models of cardiac dysfunction or pulmonary disease. Pulmonary blood volume can complement cardiac functional parameters as a further hemodynamic parameter.

## Introduction

Pulmonary blood volume (PBV) is defined as the amount of blood within the vasculature of the lung consisting of pulmonary arteries, capillaries and veins^1^. Influenced by physiological processes and pathological conditions, it represents an interesting hemodynamic parameter of the pulmonary circulation. The latter is a rather compliant circuit and serves as an important reservoir accounting for approximately 10–20% of the total body blood volume^2^. The exact amount of blood in the lungs is depending on the output of the two ventricles as well as the distensibility of the pulmonary vasculature. For the assessment of pathological processes that influence these parameters determination of the PBV can contribute valuable information. Recent clinical studies have demonstrated that the PBV serves as prognostic marker for diseases affecting the heart and/or lung, in particular chronic heart failure^3^, systemic sclerosis^4^, and acute pulmonary embolism^5^. For preclinical research in cardiac and pulmonary disease mouse models are widely used^6,7^, for which PBV may also serve as an interesting parameter. However, non-invasive measurement of PBV in mice appears to be challenging due to the animals’ small size and high heart rate.

To determine the PBV different methods have been described in the past. In principle, in vivo assessment of PBV requires measurements of the pulmonary transit time (PTT) of blood and the cardiac output^8^. The indicator dilution technique with sampling of dyes injected into the heart chambers and pulmonary circulation was originally used for this purpose^9^. Later, nuclear radiography allowed to follow injected radioisotopes within the heart^10^. With the upcoming of cardiac magnetic resonance imaging (MRI) and magnetic resonance angiography, assessment of cardiac function and first pass of a contrast agent bolus within the cardiopulmonary vasculature became feasible without radiation exposure^11^. This principle was also applied to contrast enhanced echocardiography for assessment of PBV^12^. These methods are hardly transferable to an in vivo examination of rodents, in particular mice, because of the demand of very high spatial and temporal resolution to assess cardiac function and to follow a contrast bolus within the heart and lung of a mouse. To overcome these restrictions the idea of this study was to combine two imaging modalities offering high spatial and high temporal resolution, respectively.

Magnetic particle imaging (MPI) is a new preclinical imaging modality scanning magnetic particle distributions in vivo in a three-dimensional (3D) acquisition mode with a high temporal resolution^13^. The fundamental principle of MPI can be shortly described as the nonlinear response of an injected magnetic tracer on an external sinusoidal oscillating magnetic field which is detected by a coil^14^. Its induced voltage signal is characteristic for the amount of tracer and magnetic composition^15,16^. To deduce from a measured voltage to the amount of magnetic particles in a definite volume a system matrix approach is often used^15^. To build up a system matrix a defined amount of tracer is measured as a delta probe successively at discrete positions inside the field of view using a robot. Then in Fourier space the inverse of the system matrix has to be multiplied with a vector representation of the Fourier transformed voltage signals. This numerical ill-posed system can be solved by the iterative Kaczmarz algorithm with Tikhonov regularization. It has to be mentioned that measuring the system matrix is a time consuming process but can be performed at a different time point than the experiment. Alternatively, it can be modeled knowing the magnetic properties with the drawback of not knowing nonlinearities in the hardware chain^17^. However, as MPI only provides information of the tracer distribution another auxiliary imaging modality is needed to correlate the findings to anatomy. MRI is the ideal partner modality as it can provide high spatial resolution and functional information of the cardiovascular system in mice^18,19^. Thus, the aim of this study was to establish a combined MPI-MRI approach with the use of superparamagnetic iron oxide (SPIO) nanoparticles to determine the PBV in mice as hemodynamic parameter of the pulmonary circulation.

## Materials and methods

### Animal handling

Animal experiments were carried out after approval (protocol# TVA 42/14) of the local committee for animal care and use (Behörde für Gesundheit und Verbraucherschutz, Hamburg, Germany), carried out in compliance with the ARRIVE guidelines and performed in accordance with relevant guidelines and regulations. Eight mice (Friend leukemia virus B (FVB/NCrl), female, Charles River Laboratories, Germany) with mean body weight of 26.6 g ± 1.1 g and mean age of 15 weeks were examined. Mice underwent imaging with MRI and MPI under general anesthesia with 1–2% isoflurane (l-chloro-2.2.2-trifluoroethyl difluoromethyl ether, ABBVIE, Wiesbaden, Germany) and 100% oxygen at a flow rate of 500 ml/min and a respiratory rate of about 40 breaths per minute. The respiratory cycle was observed with dedicated small animal monitoring units during MRI (SA Instruments, New York, USA) and MPI (Minerve, Esternay, France). Body temperature was kept constant by incorporated heating systems.

### Data acquisition

Measurements of cardiac function and PTT were performed on different days. Cardiac MRI measurements were carried out using a dedicated preclinical 7 T system (ClinScan, Bruker, Germany) with an 8-channel surface coil (Rapid Biomedical, Rimpar, Germany). For cardiac triggering a pulse oximeter was placed on the tail (SA Instruments, New York, USA). Cardiac triggered 2D gradient echo sequences were performed to acquire long axis and four chamber views to plan a series of short axis cine slices from the base of the left ventricle to apex of the heart. Each slice was acquired at a voxel size of 167 × 167 × 1000 µm^3^ and a temporal resolution of 5 ms resulting in an acquisition time of around two minutes (see Table 1).

**Table 1:** MRI sequence protocol parameters: GRE—gradient echo, FOV—field of view, TR—repetition time, TE—echo time, FA—flip angle, NSA—number sum averages.

Sequence	Purpose	Coil	FOV	Matrix	Slice thick-ness	Slices	TR	TE	FA	Phase partial Fourier	Phases	NSA	Bandwidth	Resolution	Scan time	Comment
		Channels/type	mm		mm		ms	ms	°				Hz/pixel	µm^3^	min:s	
Four chamber view cine GRE	Planning of short axis	8/receive	30	192 × 192	1	1	5	2.55	25	6/8	24	2	400	156 × 156 × 1000	~ 2:00	Flow compensation
Long axis cine GRE	RR length estimation	8/receive	30	192 × 192	1	1	6	2.55	25	6/8	32	2	400	156 × 156 × 1000	~ 2:00	Flow compensation
Short axis cine GRE	Heart volumetry	8/receive	32	192 × 192	1	1	6	2	25	6/8	29	2	330	167 × 167 × 1000	~ 1:40	8 times with 1 mm shift
Inflow sensitive GRE	Anatomy for fusion	1/send-receive	30	192 × 192	0.6	32	48	0.78	25	6/8	8	4	2000	156 × 156 × 600	4:15	3 times with 200 µm offset

For the pulmonary transit time measurement mice received a tail vein catheter and were positioned on an MPI-MRI-compatible mouse bed (Minerve, Esternay, France). Mice underwent MRI scans of the whole body using a volumetric body coil with a diameter of 4 cm (Bruker, Ettlingen, Germany). A series of coronal and sagittal flow sensitive gradient echo sequences depicted anatomy and in particular vessels.

Subsequently, mice were transferred to the MPI scanner (Bruker, Ettlingen, Germany). The scanner was installed nearby the MRI scanner in a small animal imaging core unit. MPI measurements were carried out with a drive field amplitude of 12 mTµ_0_^−1^ at 25 kHz and a gradient strength in z-axis of 2 Tm^−1^µ_0_^−1^ and a sampling bandwidth of 1.25 MHz. The Lissajous trajectory of the field free point covered a field of view of 24 × 24 × 12 mm^3^ within 21.5 ms resulting in a sample rate of 46 volume images per second. In order to examine the first pass through the right ventricle, the lungs and the left ventricle a sub-second bolus profile was assumed to be advantageous in comparison to a longer profile. This was accomplished by a small volume of a highly concentrated tracer, 1 µl of 1 M SPIO solution (Perimag, Micromod, Germany) followed by 50 µl saline were injected in less than one second during the performance of a dynamic measurement sequence.

### MPI reconstruction

For reconstruction of MPI data a system matrix measurement was used that was acquired before the animal experiments with the same tracer and the same hardware parameters as for the dynamic in vivo scans. During the system matrix measurement, a robot integrated in the MPI scanner (Philips/Bruker, Germany) was moving a point probe consisting of 2 µl of a 100 mM SPIO solution stepwise within 36 h at 28 × 26 × 16 positions covering a volume of 28 × 26 × 16 mm^3^.

An available framework (MPIReco.jl, www.github.com/MagneticParticleImaging) was used to reconstruct images of the 3D datasets with a voxel size of 1 × 1 × 1 mm^3^ at a temporal resolution of 21.5 ms. The reconstruction parameters^15,20^ of the iterative Kaczmarz algorithm with Tikhonov regularization were: regularization factor 0.5, iterations 5, averages 1 with frequency filtering parameters: SNR threshold 1.8, frequencies 80 kHz–1.25 MHz, and with spectral cleaning enabled.

### Image analysis

Image data processing was performed with ImageJ (NIH, USA) extended by an in-house developed plugin (qMapIt). Two independent observers identified the phases of maximal contraction and maximal dilatation of the left ventricle in the short axis cine scans and contoured the inner lumen. To calculate the ventricle volumes in systole V_systole_ and diastole V_diastole_ the areas of contoured regions were summed up and multiplied by the slice thickness. The cardiac stroke volumes were estimated by the differences between V_systole_ and V_diastole_. Furthermore, to rate the reliability interclass correlation (ICC) analysis (R version 3.6.0, package “irr”) were performed for the volumetric measures.

Images of the MRI- and MPI-examinations were co-registered and fused to identify the left and right ventricle in MPI-data. Standardized regions of interest (ROI) of 1 mm^2^ where placed in the right and left ventricle by searching for the maximal signals. Signal to time curves for each pixel were temporally smoothed by a Savitzky–Golay-filter with a width of 150 ms to average out cardiac pulsation. Signal intensities in both ventricles were analyzed by determination of the full width at half maximum (FWHM) to characterize bolus profiles. Then arrival times were determined for each pixel by fitting a sigmoid model function [see Eq. (1)] with four parameters^21^.

1 $$ signal\left( t \right) = D + \left( { A - D } \right) / \left( { 1 + \left( \frac{t}{C} \right)^{B} } \right) $$

The parameters A and D described the minimum and maximum signal intensity values of the curve and were fixed during the fitting process. Only the point of inflection C and the steepness of the curve B were optimized by a Powell’s Dogleg method. From the derived parameters, a linearization around the inflection point was developed and the intersection position with the function signal(t) = A was calculated. This position reflects the arrival time, the first appearance of the tracer bolus in the time course of a voxel. Then the time of the bolus arrival t_0_ was calculated by the root of a line with the derivative in the point of inflection crossing the curve in C. To calculate the error of t_0_ a covariance analysis was performed to derive the standard deviation of B and C which were used in the error propagation.

Next, the difference of arrival times in the two regions of interest located in the right and left ventricle were determined. This time difference represented the PTT of the bolus through the lungs. The length of RR-interval (RR) was determined by Fourier analyzing the signal modulation of blood in the left ventricle by finding the frequency with the maximal amplitude. These MPI based values were later on compared to the RR-interval length that was derived from cine MRI of the heart by counting the phases between two successive maximal contractions of the left ventricle. Using the MPI based parameters of PTT and RR-interval as well as MRI derived parameters V_systole_ and V_diastole_ the lung blood volume V_lung blood_ was calculated [see Eq. (2)] according to the following equation similar to the one of Stewart el al.^8^:

2 $$ {\text{V}}_{{{\text{lung}}\;{\text{blood}}}} = \frac{{{\text{V}}_{{{\text{stroke}}\;{\text{volume}}}} }}{RR} \cdot PTT = \frac{{{\text{V}}_{{{\text{distole}}}} - {\text{V}}_{{{\text{systole}}}} }}{RR } \cdot PTT $$

As propagations of errors of each measurement procedure occur this was specifically addressed. For simplicity the maximal error limit $\Delta {\text{V}}_{{{\text{lung}}\;{\text{blood}}}}$ was calculated by the maximal error limits of each measurement step as seen in Eq. (3). The error limits for ventricle volumes $\Delta {\text{V}}_{{{\text{diastole}}}}$ and $\Delta {\text{V}}_{{{\text{systole}}}}$ were assumed to be 5% of their mean volumes, as the error of RR determination $\Delta RR$ was assumed to be 3%. The transit time error limit $\Delta PTT$ was calculated by adding the standard deviations of the arrival times in the ROIs and the standard deviation derived from the covariance analysis of the fitting procedure.

3 $$ \Delta {\text{V}}_{{{\text{lung}}\;{\text{blood}}}} = \Delta {\text{V}}_{{{\text{stroke}}\;{\text{volume}}}} + \Delta {\text{V}}_{{{\text{transit}}\;{\text{time}}}} + \Delta {\text{V}}_{{{\text{RR}}\;{\text{length}}}} $$

$$ \Delta {\text{V}}_{{{\text{stroke}}\;{\text{volume}}}} = \frac{PTT }{{RR }} \cdot \left( {\Delta {\text{V}}_{{{\text{diastole}}}} + \Delta {\text{V}}_{{{\text{systole}}}} } \right) $$

$$ \Delta {\text{V}}_{{{\text{transit}}\;{\text{time}}}} = \frac{{{\text{V}}_{{{\text{diastole}}}} - {\text{V}}_{{{\text{systole}}}} }}{RR } \cdot \Delta PTT $$

$$ \Delta {\text{V}}_{{{\text{RR}}\;{\text{length}}}} = \frac{{{\text{V}}_{{{\text{diastole}}}} - {\text{V}}_{{{\text{systole}}}} }}{RR } \cdot PTT \cdot \Delta RR $$

All reported measured values were given as mean values and standard deviation (mean ± SD).

## Results

**Figure 1:**
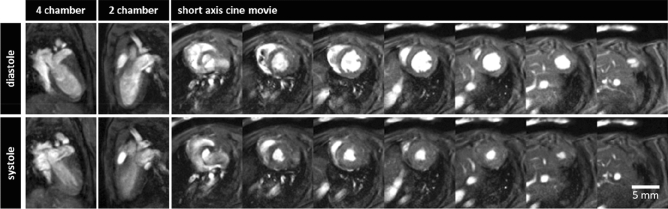
Cine sequences of the heart of a mouse in diastole and systole: four chamber view, two chamber view and a series of short axis views of the left ventricle used for volumetric analysis.

Cardiac MRI examinations provided a good image quality for assessment of ventricular function in all mice (Fig. 1). Volumetric analysis of the short axis cine views revealed a mean left ventricular volume in diastole of 45.2 µl ± 5.6 µl with an ICC of 0.94 and in systole of 13.5 µl ± 4.3 µl with an ICC of 0.97. The stroke volume was 31.7 µl ± 2.3 µl with an ICC of 0.70 with an ejection fraction of 71% ± 6% with an ICC of 0.94 at a RR-interval length of 133 ms ± 7 ms.

**Figure 2:**
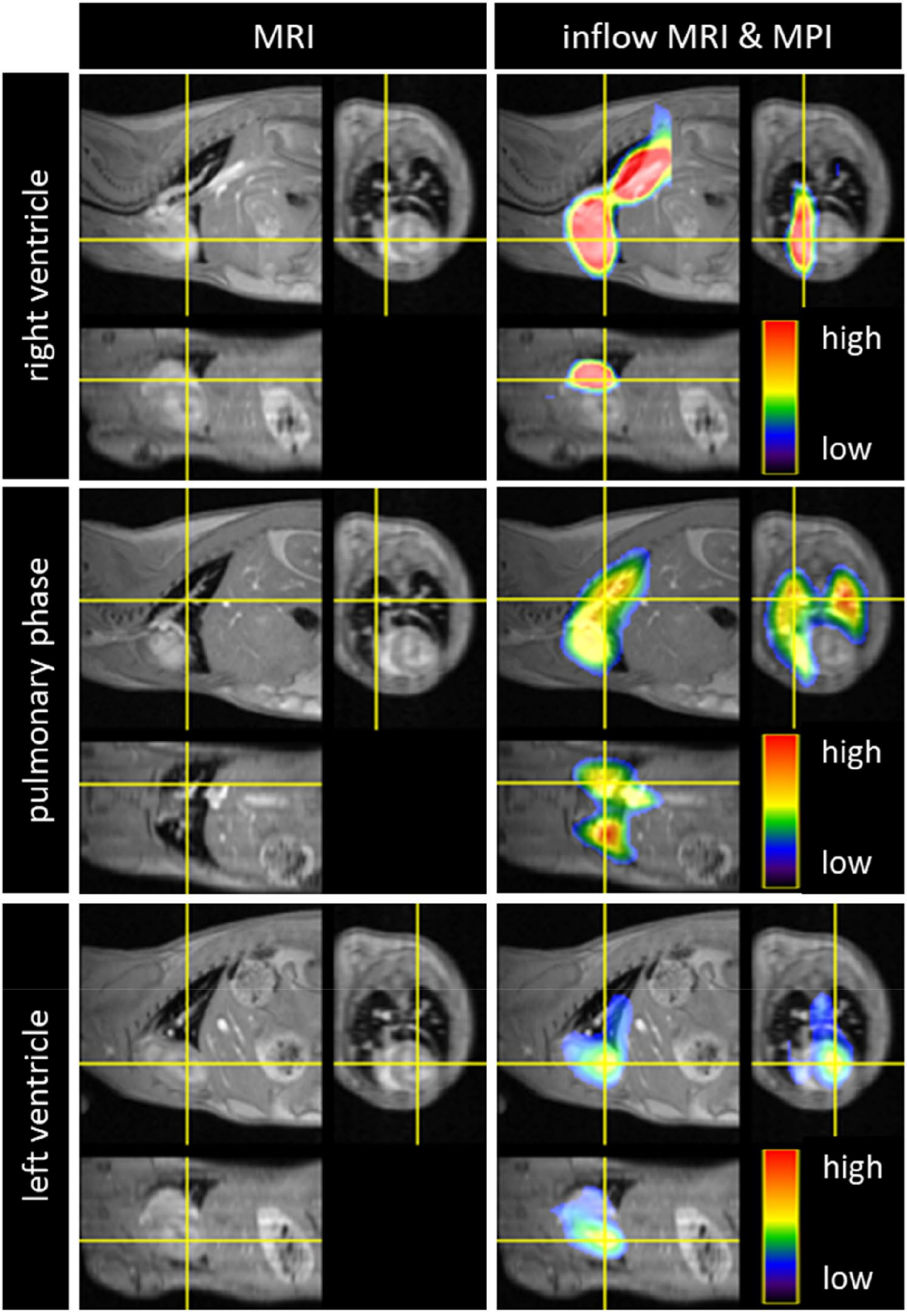
MRI and fused MPI-MRI-data at three distinct time points showing the inflow of tracer into the right ventricle, the passage through the lungs and the arrival at the left ventricle. The MPI signal intensities are color coded. A high amount of tracer per pixel is indicated in red while a low amount in blue.

**Figure 3:**
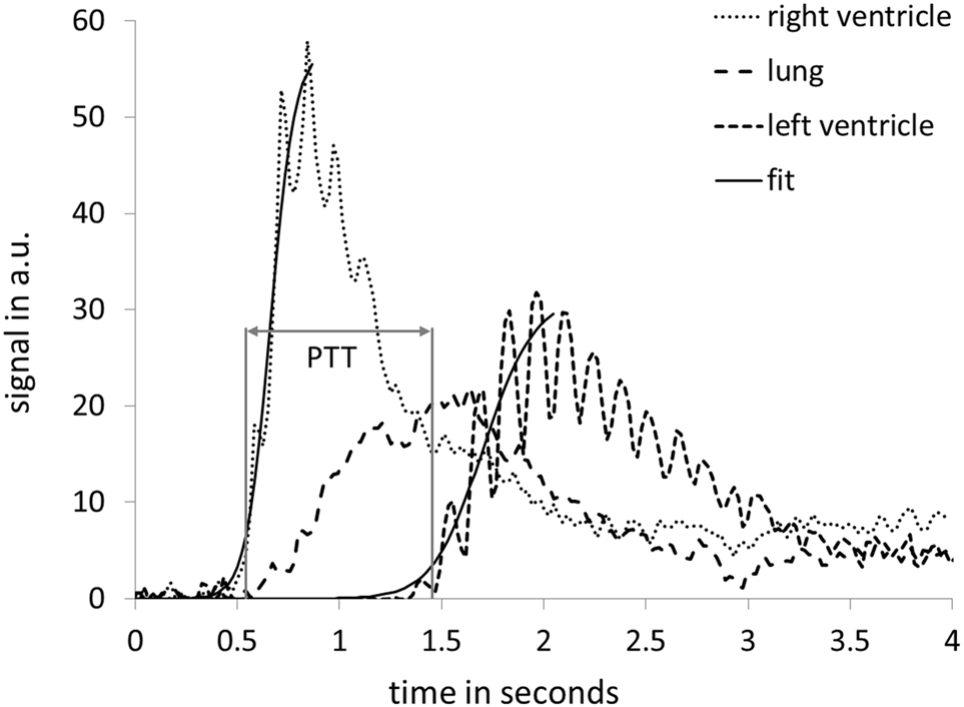
First pass in three phases: the signal time curves measured by magnetic particle imaging show a change in intensities caused by the inflow of tracer into the right and left ventricle as well as in the lungs of a mouse. The three curves are temporally separated due to the sharp bolus profile in the right ventricle. The pulmonary transit time (PTT) describes the delayed arrival in the left ventricle. The observed signal modulation in the left ventricle with a frequency of 7.2 Hz was caused by the cardiac cycle with a RR-interval length of 139 ms.

**Figure 4:**
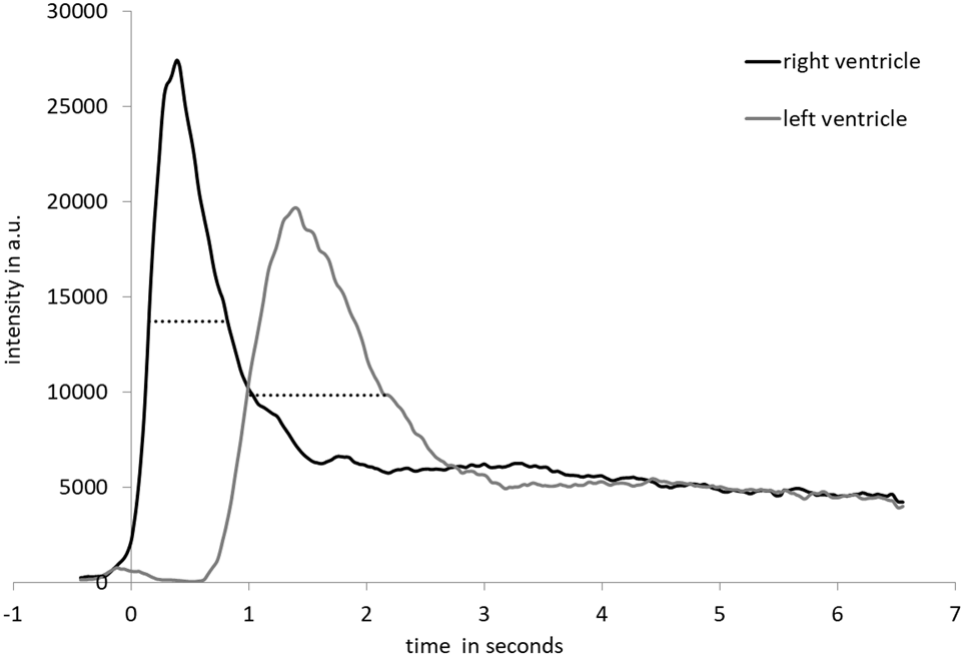
Short bolus profile in the right ventricle and dispersion in the left ventricle measured by magnetic particle imaging: averaged signal to time curves of eight mice show a full width at half maximum of 578 ms ± 144 ms in the right ventricle and 1042 ms ± 150 ms in the left ventricle. After 4 s signal intensities are even.

**Figure 5:**
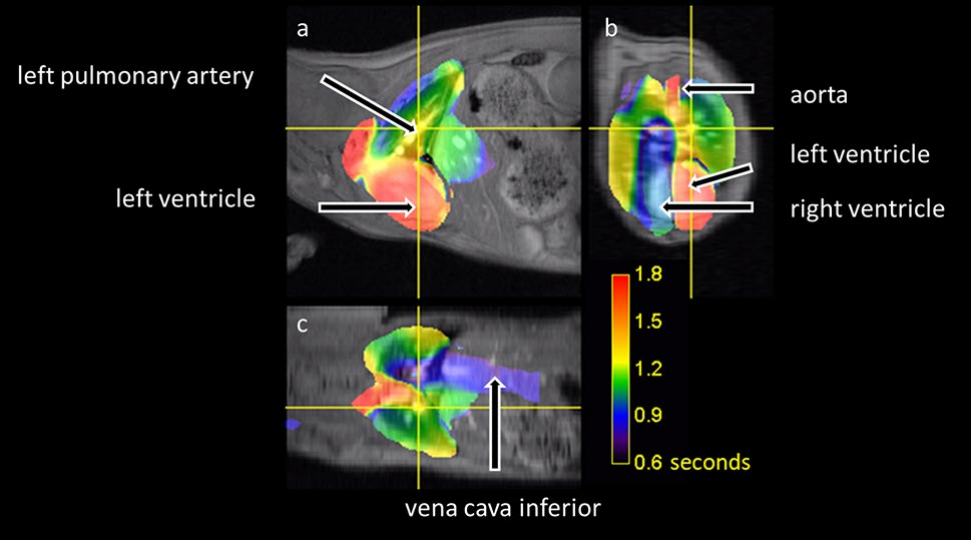
Bolus arrival time map fused with MRI in (**a**) sagittal, (**b**) transversal and (**c**) coronal perspective. The presence of the tracer bolus in the right ventricle starting at about 0.9 s is shown in blue colors and in the left ventricle appears approximately 0.8 s later in red color. Lung perfusion is shown in green and yellow colors representing the time interval in between.

**Figure 6:**
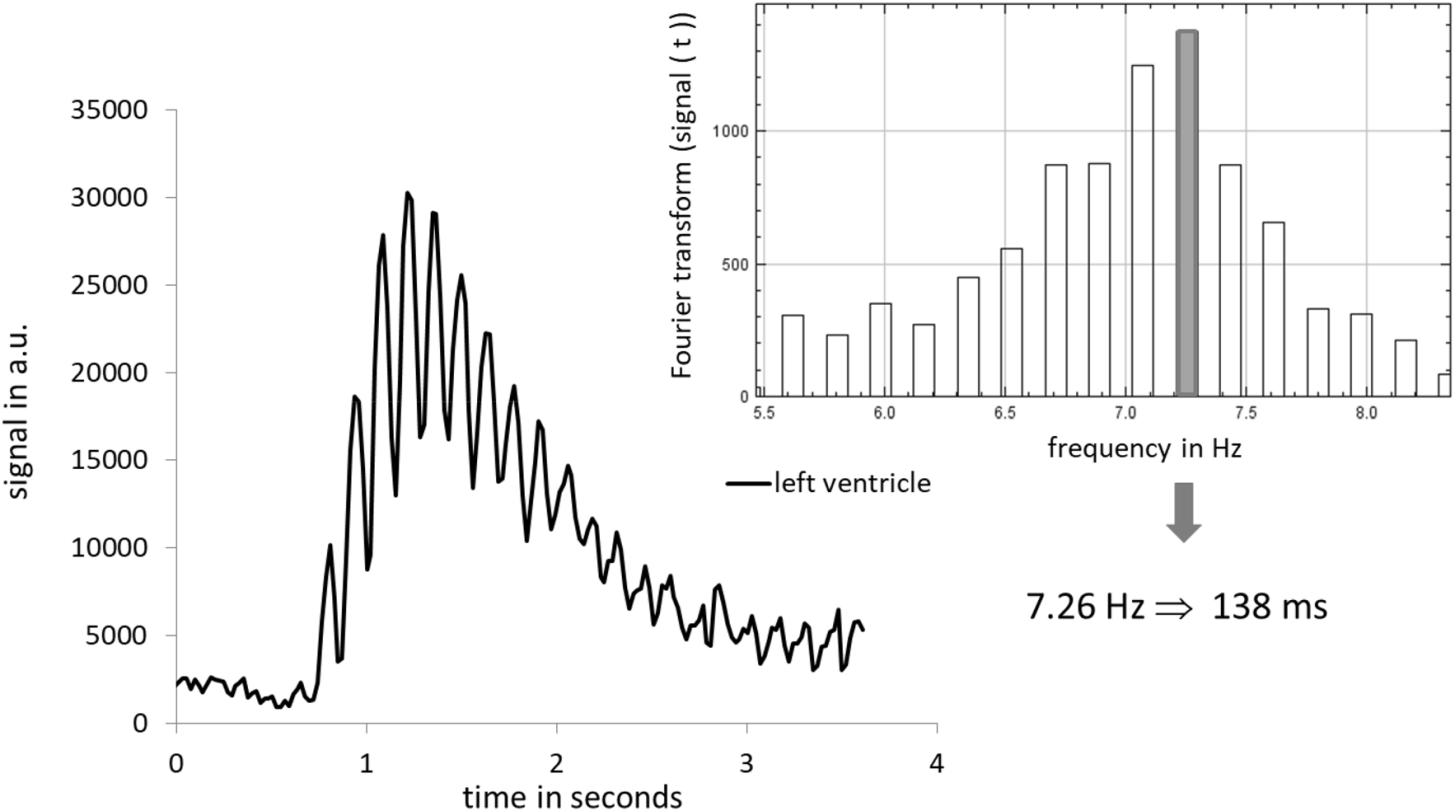
Estimation the RR interval length from MPI signal modulation in the left ventricle of a mouse by a Fourier analysis of the most pronounced frequency amplitude.

**Figure 7:**
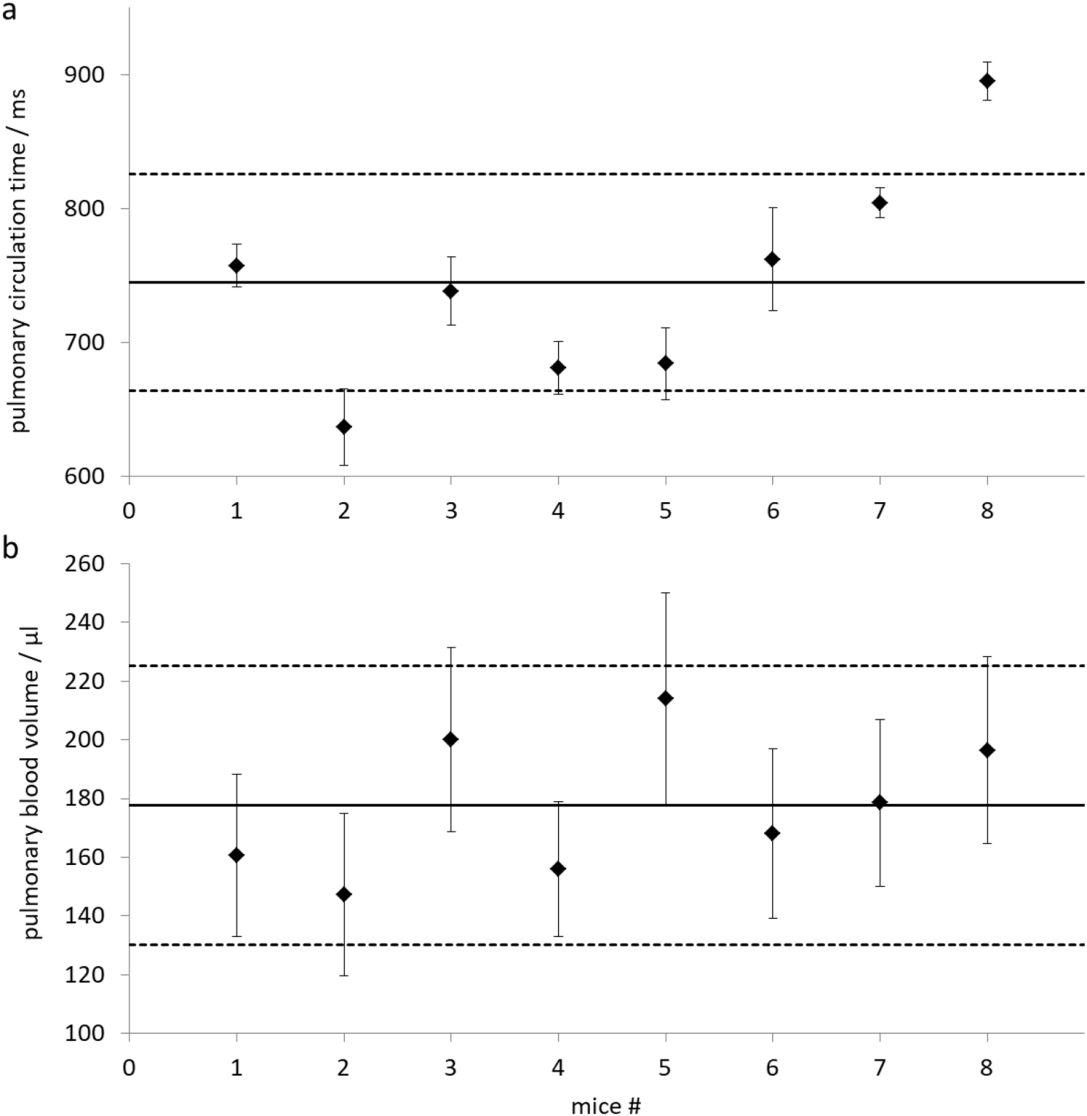
(**a**) Pulmonary transit times and (**b**) pulmonary blood volume in eight mice. Error bars indicate the standard error and the dashed lines the 95% confidence intervals around the mean values of around 745 ms and 177 µl.

MPI examinations and co-registrations with morphological MRI data were successful in all mice. The SPIO bolus could be observed throughout the heart and pulmonary vessels (Fig. 2). The signal to time course allowed separating the first pass into three distinct phases representing the tracer’s arrival in the right ventricle, the pulmonary vasculature and the left ventricle (Fig. 3). The profiles of the SPIO bolus in the right and left ventricles were further characterized. The FWHM of the bolus in the right ventricle was 578 ms ± 144 ms and 1042 ms ± 150 ms in the left ventricle while reducing its signal strength by around 42 percent (Fig. 4). The mean time difference between bolus arrivals in the right and left ventricles and thus the PTT was 745 ms ± 81 ms. Time difference between the maxima was prolonged to 1033 ms ± 210 ms. The difference in the arrival times can be displayed by fusing an arrival time map with the morphological MRI data (Fig. 5). The mean RR-interval length derived by Fourier analysis of the cardiac induced signal modulation (Fig. 6) revealed 133.4 ms ± 11.5 ms. The following calculation of PBV resulted in a range of 152–207 µl (Fig. 7) with a mean PBV of 177 µl ± 27 µl .

The maximal error limits for each PBV value ranged between 22 µl and 38 µl with a mean of 27.0 µl ± 5.2 µl which is a relative maximal error of 15 percent. The major contributions to the maximal error limits were given by the two volumetric assessments of the left ventricle whose error limits propagated to be 16.4 µl disclosing the importance of these measurements. The maximal error limit for PTT was 22.6 ms which results in additional 5.3 µl and another 5.3 µl for measurement of RR-interval length error of 4 ms.

## Discussion

In this study the determination of PBV in mice was investigated utilizing two imaging techniques. These were used to analyze the passage of SPIO nanoparticles traveling from the right ventricle through the lungs to the left ventricle as well as to determine cardiac function.

Due to its fast data acquisition MPI can resolve the first pass of the tracer through the lung in a 3D imaging mode with a temporal resolution of 21.5 ms without triggering vital signals. MPI is a rather new imaging technology that is available for preclinical imaging only. A first clinical demonstrator was presented being feasible to scan a head phantom^22^. Its advantages are that it is acquiring data in 3D and is able to measure data in real time without ionizing radiation. However, MPI’s spatial resolution cannot yet compete with that of MRI as demonstrated by an angiography of ex vivo pig kidneys^23^. In future, provided that spatial resolution has improved, cardiac functional volumetry and pulmonary blood volume measurement in a one-stop-shop MPI protocol could become feasible. High gradient strength increases the resolution on the cost of a restricted field of view which makes it for the operator more challenging not to miss the target region. To compensate for this the drive field amplitude may be increased on the cost of a higher specific absorption rate. The consequence of all is a higher energy consumption and the need of more cooling hardware.

In our experimental configuration MRI’s spatial resolution is around ten times higher compared to MPI. Thus, the accurate assessment of the cardiac stroke volume was performed by cardiac MRI. Combing the results from MPI and MRI, in detail cardiac stroke volume, length of RR-interval, and PTT; the PBV could be calculated. To our knowledge this is the first study demonstrating noninvasive in vivo assessment of PBV in mice.

Zarbock et al.^24^ estimated invasively the PBV in wild type and Alox15 mice by ligating the pulmonary vessels five minutes after an Evan blue injection and scarifying animals. By measuring volumes of the extracted lung and concentrations of the dye in lung and blood the authors were able to depict the pulmonary blood volumes in wild type mice to be 141 µl ± 13 µl and in Alox15 mice to be 153 µl ± 21 µl. These values are lower than our observed values of 177 µl in FVB mice. One reason for the derivation of around 30 µl may be caused by differences in age, body weight and mouse strains. However, this does not satisfactorily explain this rather large disagreement. Instead variabilities of the different measurement procedures seem to be more crucial. On the one hand, we measured the arrival time of a tracer bolus in the left ventricle instead of the left atrium and therefore overestimated the PTT as blood is passing an additional albeit small cardiac chamber. On the other hand, ligating pulmonary vessels leads to shortening of vascular structures and hence the vascular volume. Therefore, it appears reasonable that the true PBV of mice at rest corresponds to a volume between the values of these different measurement techniques.

The main advantage of our measurement procedure is its noninvasiveness and therefore the possibility to repeat this examination for longitudinal studies. Furthermore, additional cardiovascular parameters of interest, in particular the cardiac stroke volume and PTT, are determined as well. The latter also represents a valuable parameter of pulmonary hemodynamics. Different clinical studies have demonstrated a prolonged PTT in association with increased pulmonary arterial pressure (PAP), increased pulmonary capillary wedge pressure (PCWP) and decreased left ventricular function in patients with pulmonary arterial hypertension^25^, heart failure^26,27^ and congenital heart disease^28^.

Nevertheless, evaluation of PTT in mice is rather difficult due to the high heart rate. In 2006 Kreissl and colleagues assessed cardiovascular function in mice noninvasively by high temporal resolution small animal PET and estimated the PTT in the range of one second^19^. However, temporal resolution of the micro-PET was limited to three frames per second and prevented further analysis of this parameter. Later, Sonobe et al. investigated the pulmonary circulation of mice by synchrotron radiation microangiography with injection of an iodine contrast agent into the right ventricle via a transjugular microcatheter^29^. They were able to acquire image data at a rate of 30 frames per second und thus could assess PTT between the right and left ventricle at rest with a mean value of 0.83 s ± 0.03 s, which is in the range of our observations. While this method with an intracardiac microcatheter allowed acquiring further information of the pulmonary circulation, such as right ventricular systolic pressure, its invasiveness also represents the major drawback of this technique as right heart catheterization in mice is considered to be a final procedure before sacrifice. Recently, a preclinical study with noninvasive assessment of PTT in mice by 4D optoacoustic imaging was published. Lin et al. performed tomographic optoacoustic ultrasound measurements with a temporal resolution of 50 volumes per second in infarcted and healthy mice and found a PTT of 2.25 s and 1.34 s, respectively^30^. Although optoacoustic ultrasound and MPI have a similar temporal resolution the PTT of 745 ms determined at our observations was shorter and thus comparable to the results of Sonobe et al^29^. The reason for this difference may have again various causes, such as differences in mouse strain, body weight and ages. An advantage of our approach over optoacoustic imaging is that the examination is not limited by light absorbing hair and the positioning procedure is simple.

However, a crucial point of the injection based methods may be also the sharpness of the signal change induced by the contrast agent or tracer. The FWHM of the bolus in our study was 578 ms and we determined the PTT by analyzing the arrival times of the bolus in both ventricles. In contrast, the bolus profile in the study of Lin et al. showed a much broader profile of around 2 s and PTT was determined as time difference of the less distinct maximum values in the right and left ventricles. We can see in our data that measuring the PTT by the time difference between the maximal values in the ventricles prolongs the time significantly to 1033 ms. We assume that measuring the transit time by the arrival times is the more accurate approach.

A limitation of our study was the fact that cardiac MRI and MPI measurements were not performed on the same day. This was due to the use of different receiver coils for cardiac MRI and whole-body MRI of mice, respectively. MRI coils were not MPI compatible and therefore had to be removed for the MPI scan. While this was easily achievable for the volumetric mouse body coil it was not possible for the 8-channel receiver coil used for cardiac MRI. The latter was fixed to the mouse bed. Changing the coil and mouse bed would have been an additional temporal burden and would have carried out the risk to dislocate the catheter in the tail vein. Consequently, cardiac MRI and MPI examinations were carried out on different days assuming that cardiac stroke volumes are rather constant under the same experimental conditions as shown by Kreissl et al.^31^. This assumption was supported by the fact that the length of RR-intervals determined by cardiac MRI and MPI were similar indicating a similar depth of anesthesia and cardiac function during the different examinations.

In conclusion, in vivo assessment of the PBV in mice was feasible using a combined MPI-MRI approach. MPI examinations with 3D acquisition in high temporal resolution enabled an accurate determination of the PTT of a contrast bolus through the mouse lung as well as of the length of the RR-interval. Cardiac MRI provided high resolution image data of the mouse heart for precise assessment of the cardiac stroke volume. Based on these data PBV could be calculated with an acceptable range of errors. Therefore, this technique represents a reliable means to assess the PBV in mice. It may be of specific value for evaluation of pulmonary hemodynamics in mouse models of cardiac dysfunction or pulmonary disease.

## Data Availability

Raw data and processed image data is available to share on reasonable request.
